# DDT and Malaria Prevention: Addressing the Paradox

**DOI:** 10.1289/ehp.1002127

**Published:** 2011-01-18

**Authors:** Hindrik Bouwman, Henk van den Berg, Henrik Kylin

**Affiliations:** 1 School of Environmental Sciences and Development, North-West University, Potchefstroom, South Africa; 2 Laboratory of Entomology, Wageningen University, Wageningen, the Netherlands; 3 Department of Water and Environmental Studies, Linköping University, Linköping, Sweden

**Keywords:** effects, health, indoor residual spraying, malaria vector management, precaution

## Abstract

**Background:**

The debate regarding dichlorodiphenyltrichloroethane (DDT) in malaria prevention and human health is polarized and can be classified into three positions: anti-DDT, centrist-DDT, pro-DDT.

**Objective:**

We attempted to arrive at a synthesis by matching a series of questions on the use of DDT for indoor residual spraying (IRS) with literature and insights, and to identify options and opportunities.

**Discussion:**

Overall, community health is significantly improved through all available malaria control measures, which include IRS with DDT. Is DDT “good”? Yes, because it has saved many lives. Is DDT safe as used in IRS? Recent publications have increasingly raised concerns about the health implications of DDT. Therefore, an unqualified statement that DDT used in IRS is safe is untenable. Are inhabitants and applicators exposed? Yes, and to high levels. Should DDT be used? The fact that DDT is “good” because it saves lives, and “not safe” because it has health and environmental consequences, raises ethical issues. The evidence of adverse human health effects due to DDT is mounting. However, under certain circumstances, malaria control using DDT cannot yet be halted. Therefore, the continued use of DDT poses a paradox recognized by a centrist-DDT position. At the very least, it is now time to invoke precaution. Precautionary actions could include use and exposure reduction.

**Conclusions:**

There are situations where DDT will provide the best achievable health benefit, but maintaining that DDT is safe ignores the cumulative indications of many studies. In such situations, addressing the paradox from a centrist-DDT position and invoking precaution will help design choices for healthier lives.

Recent publications and correspondence regarding human health implications of dichlorodiphenyltrichloroethane (DDT) in this journal (e.g., Blair et al. 2009; Burton 2009; Tren and Roberts 2010; van den Berg 2009) are evidence of a continuing debate on a subject pertinent to millions of people in three continents and some islands. The debate is polarized and could be characterized by three viewpoints that are at odds over fundamental and pragmatic issues:

- The anti-DDT viewpoint wants to eliminate any production and use of DDT because of environmental and health concerns. We could find no current outright anti-DDT activities except news from a court case in Uganda against the use of DDT for malaria control (Lewis 2008).
- The centrist-DDT point of view adopts an approach that pragmatically accepts the current need for DDT to combat malaria transmission using indoor residual spraying (IRS) but at the same time recognizes the risks inherent in using a toxic chemical in the immediate residential environment of millions of people. The continued use of DDT is strongly qualified by an urgent call from the Stockholm Convention for alternative chemicals, products, and strategies. This call inherently implies the eventual termination of DDT used in IRS for malaria control (Steiner 2009).
- The pro-DDT viewpoint considers DDT safe to use in IRS when applied correctly and promotes DDT to be used as IRS in malaria control where it is still effective. Even if eventually human health effects are found to be caused by DDT, these effects would be far less than those caused by malaria (Africa Fighting Malaria 2010; Roberts et al. 1997).

This is a simplistic outlay of the current gradient of the debate—there will undoubtedly be other ways of characterizing it—but, in broad terms, these statements reflect different views of common considerations.

## Objectives

Our objective in this commentary was to match a series of questions on the use of DDT in IRS with the literature, experiences, and insights that may not be published in scientific articles or that may not always be recognized when considering the health implications. We attempted to arrive at a synthesis and to place it along the gradient outlined above. We identified some options and opportunities to achieve safe and effective malaria control.

## Discussion

### Is DDT “good”?

DDT is used in IRS by spraying indoor surfaces with a coating of DDT. This residual coating prevents malaria transmission as a spatial repellent or contact irritant or by killing mosquitoes (indicating more than one mode of action), effectively preventing or interrupting transmission (Grieco et al. 2007). For more than six decades, DDT used in IRS for malaria control has protected the lives of millions of people and prevented the suffering of millions more across the globe [estimated from Knipling (1953) and Mabaso et al. (2004)]. For example, when DDT was replaced with alternative chemicals for IRS in South Africa, the number of cases and deaths from malaria increased suddenly. The reintroduction of DDT (among other new measures) halted and reversed this epidemic, strongly indicating the effectiveness of DDT for IRS (Mabaso et al. 2004; Maharaj et al. 2005; Sadasivaiah et al. 2007). Seen from this defined perspective, DDT must be seen as “good,” and there can be few arguments about this.

### Is DDT safe under IRS conditions?

DDT is a chemical specifically made and used to kill living things—its toxicity is indicated on all labels. With multiple modes of action, it is particularly effective against insects and was used in large quantities in agriculture and public health [World Health Organization (WHO) 1979]. However, the biological activity of DDT is not limited to insect biochemical systems. Over decades, DDT has been associated with effects, as have countless other chemicals, on a number of noninsect biological systems, including humans (Eskenazi et al. 2009).

End points such as those associated with endocrine disruption (Longnecker et al. 2007), neurological development (Eskenazi et al. 2006), and behavior (Ribas-Fitó et al. 2006) have more recently been deployed, enabling investigations in addition to cancer (Cohn et al. 2007), childhood growth (Karmaus et al. 2002), and alteration of enzyme function (Bouwman et al. 1991). The Pine River Statement reviewed 494 studies published between 2003 and 2008 and found that “DDT and its breakdown product DDE [dichlorodiphenyldichloroethylene] may be associated with adverse health outcomes such as breast cancer, diabetes, decreased semen quality, spontaneous abortion, and impaired neurodevelopment in children” (Eskenazi et al. 2009). However, very few studies were available for the Pine River Statement from countries using DDT for malaria control.

Since the publication of the Pine River Statement, more studies on human health effects have been published; some of these have been from developing countries. We conducted a search of PubMed (National Library of Medicine, Bethesda, MD, USA) for studies published in 2009 using the same search terms as the Pine River Statement (Eskenazi et al. 2009). [For a short overview of the findings, see Supplemental Material (doi:10.1289/ehp.1002127).] Of the 22 epidemiological reports on human health effects, 9 showed no significant associations between DDT or DDT and DDE and effects (e.g., some serum hormone levels, breast cancer, or childhood leukemia), and 12 showed significant associations of DDT and DDE with conditions or effects on type 2 diabetes, hormones in blood, infant birth weight, pancreatic ductal adrenocarcinoma, and sperm parameters. One report did not investigate specific outcomes. It must be noted that many of the European, Japanese, and American studies reported primarily on *p*,*p*′-DDE levels, because this metabolite is the main legacy of past *p*,*p*′-DDT applications. In malaria-controlled areas, *p*,*p*′- and *o*,*p*′-isomers of DDT, DDE, and dichlorodiphenyldichloroethane (DDD) are all present, each with their own toxicities (e.g., Gelbke et al. 2007; Kang et al. 2004; Kelce et al. 1995; Leavens et al. 2002; Shin et al. 2007) and environmental chemical behaviors (e.g., Bouwman et al. 2006; Okonkwo et al. 2008; Sereda et al. 2009; van Dyk et al. 2010), and often at much higher levels than non-IRS exposure scenarios.

Combined with the Pine River Statement and other assessments (e.g., Longnecker 2005; Sadasivaiah et al. 2007), these continued findings and observations of associations of DDT with human health effects [see Supplemental Material (doi:10.1289/ehp.1002127)] cannot be disregarded. Given the current evidence, an unqualified statement that DDT as used in IRS is safe is untenable.

### How are inhabitants exposed?

DDT applied as IRS continuously exposes all members of a household, including infants, children, pregnant mothers, and the elderly. At applications of about 64–128 g/year per dwelling (mean wall area, 32–64 m^2^ at 2 g/m^2^), DDT is continuously bioavailable in the homestead because it has to remain effective against mosquitoes. An assessment of how inhabitants take up DDT should take into account all exposure scenarios and routes of uptake around a homestead. Analogous to the “total indoor environment” approach advanced by Lioy (2006), the “total homestead environment approach” (THEA) has been advanced (Sereda et al. 2009; van Dyk et al. 2010). THEA not only includes the indoor aspects (e.g. levels of chemicals in air and on surfaces) but also recognizes the diverse outdoor activities associated with homestead living in many developing countries in the immediate vicinity of the homestead (e.g., cooking, sleeping, lounging, home gardening, playing). THEA also establishes an indoor–outdoor link, such as sweeping and cleaning, and the movement of domestic animals. In a study conducted by van Dyk et al. (2010), high levels of DDT were found in soil immediately outside homesteads, as well as in chickens, likely due to regular sweeping of DDT-laden dust to the outdoors.

Sereda et al. (2009) postulated that a continuous process of indoor sublimation, revolatilization, and deposition of DDT, as well as movement via dust resulted in a redistribution of DDT throughout the dwelling and outside. Their findings indicate that DDT does not remain only on applied surfaces. The spatial repellent effect of DDT is obvious from mosquitoes exiting or even avoiding DDT-sprayed dwellings (Grieco et al. 2007). For DDT to have that sort of effect implies that DDT is in the air inside and perhaps even outside the dwelling. Evidence that DDT is chronically present in air was shown recently by van Dyk et al. (2010): DDT remained detectable in indoor air for at least 84 days after application. This further implies continuous exposure by all inhabitants to airborne DDT. THEA has therefore shown multiple routes of exposure and uptake.

Reported DDT levels in breast milk often exceed the tolerable daily intake of and maximum residue limits in dairy milk for adults (Bouwman et al. 1990, 2006; Okonkwo et al. 2008). Although most breast milk studies that report DDT levels acknowledge that breast-feeding should be continued (Bouwman et al. 2006; Okonkwo et al. 2008), it is not a tolerable situation. Levels such as these in any developed country would cause great concern and action. A globally shared responsibility implies the acceptance of a shared, not differentiated, value system.

IRS workers are also exposed, of course. In many instances, prescribed personal protection procedures and safe practices are not followed, because of uncomfortable working conditions. However, IRS workers are exposed to DDT on a daily basis throughout the spraying season when mixing formulations, loading canisters, and applying DDT. Not wearing masks or gloves and frequent wiping of sweaty faces with the same cloth (Bouwman H, unpublished data) increases dermal and inhalation uptake. Observations of these practices indicate very high exposure. Serum levels in IRS workers in South Africa were indeed high compared with the general population living in DDT-sprayed houses (Bouwman et al. 1991). On the other hand, Bimenya et al. (2010) showed hardly any increase in serum DDT over an entire spray season in Ugandan DDT applicators and ascribed this to protective clothing and strict adherence to WHO (2000) guidelines. Their findings demonstrate that effective exposure reduction is possible.

### Other DDT-associated impacts from malaria areas?

As for human health, research on DDT-associated impacts on biota from malaria controlled areas is scarce. Barnhoorn et al. (2009) found DDT in fish, and Marchand et al. (2008) and Barnhoorn et al. (2010) found indications of endocrine disruption in fish, all in the same major river that flows through a DDT-sprayed (IRS) region of South Africa, but both investigations were unable to establish that DDT was the cause. In the same region, rates of urogenital malformations in baby boys were elevated compared with areas where DDT was not used [Bornman et al. 2010; see also Supplemental Material (doi:10.1289/ehp.1002127)]. Laboratory tests with DDT on fish showed decreased survival, skeletal deformities, increased oocyte atresia in ovaries, and disorganization of seminiferous tubules (Mlambo et al. 2009). More studies on birds, fish, and snails in this region are ongoing.

### Should DDT be used?

Overall, community health is significantly improved through the many malaria control measures, which include IRS with DDT. Several investigators who are pro-DDT have advocated for the continued use of DDT:

- “Use of this insecticide [DDT] should not be abandoned unless its known detrimental health effects are greater than the effects of uncontrolled malaria on human health” (Roberts et al. 1997).
- “In addition, the benefits of its [DDT] use are far greater than any supposed negative human health impacts and, because the benefits are felt immediately, whereas any potential negative impacts will take place in the future, DDT passes the PP [precautionary principle] test” (Tren and Bate 2001).
- “Even if the many studies on DDT do eventually conclude that there is some proven human health harm from DDT, that risk would still have to be balanced against the risks from malaria” (Africa Fighting Malaria 2010).

However, the evidence of adverse health effects due to DDT (as for many other chemicals) is mounting as more research is published (as discussed in the preceding section). This new research clearly indicates that it is now time to, at the very least, invoke precaution on the use of DDT in malaria control. Clearly, protecting lives is the priority, but who will take care of those that are protected but harmed? And what can be done to reduce exposure? Developing safe and effective alternatives to DDT would be a major step forward.

The mounting evidence of a DDT-associated health burden should not be ignored, even if such a health burden does not nearly equate with malaria morbidity and mortality. Advancing an argument that DDT should be continued because DDT’s negative effects are so much less than are the effects of malaria on mortality or morbidity, and then ending the discussion there, ignores the rights of people to a safe environment, or at least to live safely in a compromised environment. The situation can be compared with taking prescription medicine. Prescribing a drug for a malady is expected to improve or eliminate the malady, but there are possible side effects. Patients are informed of these side effects, and actions can be taken if required. These are ethical obligations of medical treatment currently not very prominent in the communication of the public health use of toxic chemicals. Much more attention should therefore be focused on informing the public about the advantages of malaria control, even if this still requires DDT, and ways and means to reduce exposures. Also, if communities suffer effects at rates above the norm, and the existing health support infrastructure is not equipped to recognize or deal with it, additional and targeted support systems must be contemplated once the extent of the need has been established through research.

Hence, DDT is both “good” because it saves lives, and “not safe” because it has health and environmental consequences. This creates a paradox that needs to be resolved. Malaria control cannot be halted—and in certain situations, IRS with DDT remains the method of choice. This paradox is recognized by a centrist-DDT position.

### Can DDT use be improved?

In the absence of a paradox, one can only imagine the outcry that would follow if people in the developed world were forced to have 2 g/m^2^ DDT applied to their inner residential walls once a year. Nobody wants to expose their babies to DDT via breast milk as mothers have to in DDT-treated areas. The very fact that so many precautions are built into the WHO guidelines shows that IRS chemicals are considered hazardous. Therefore, it is a matter of urgency that exposure to and uptake of DDT by inhabitants and applicators should be reduced as much as possible. IRS procedures should be improved and adhered to, and new procedures could be developed to minimize insecticide use while ensuring proper coverage with as little nontarget contamination as possible. Quality control and audit schemes can be applied to prescribed procedures, training, equipment, and consumables in ways that will also encourage and incentivize adherence to all procedures by the spray teams and management. Measures need also to be found to improve applicator protection without encumbering the applicators. Equipment checks and calibration, better facilities for mixing and pouring, and maintaining good relations with inhabitants are of great importance. All this most likely implies more research, equipment, finances, and labor (and bureaucracy).

### Can DDT be used differently?

Because DDT has more than one mode of action on mosquitoes, new and innovative ways could be investigated to achieve effective control with DDT while significantly reducing human exposure and leakage to the environment. A number of projects on alternatives to DDT are also under way (e.g., Stockholm Convention 2010). Alternatives should be implemented in the context of the strategy of integrated vector management (van den Berg 2009) that could include use of DDT.

### Opportunities

Invoking precaution may be an opportunity to revisit the active ingredient composition. Current formulations have a significant proportion of the endocrine-active *o*,*p*′-DDT component. Assuming that *o*,*p*′-DDT is not responsible for one or more of the desired modes of action, it could be reduced or eliminated. Less DDT need then be applied, resulting in reduced human exposure. The other components of DDT formulations should also be considered, because some of these may also have health implications.

The concept of THEA must be further explored. A good understanding of the dynamics of DDT and any other chemical in and around homesteads will help identify risk and exposure reduction opportunities. Toddlers and children spend much time on the ground, which has high concentrations of DDT (van Dyk et al. 2010), where they presumably inhale and ingest more dust than do people who are standing upright (Lunder et al. 2010). Efforts to reduce this exposure should concentrate on reducing pollution of the soil in and around homes. Implementing risk reduction strategies would also benefit those most likely to have the greatest risk of effects—pregnant mothers, fetuses, and babies (Bouwman and Kylin 2009). Pregnant and breast-feeding mothers are probably of the highest concern because the circulating DDT inevitably exposes the fetus and baby during their most susceptible developmental phases (Bouwman and Kylin 2009; Ostrea et al. 2009; Sapbamrer et al. 2008). Any means of achieving reduction of maternal exposure would inevitably also reduce exposures to the other members of the household—the effectiveness of these interventions should be followed by targeted research.

What can be learned from THEA and other exposure and health investigations can also be applied to emergency and developing situations. Natural disasters can lead to refugees and displaced persons becoming exposed to malaria or other vector-borne diseases. Climate change, urbanization, and environmental degradation may result in the redistribution of disease or disease vectors. Implementing the THEA lessons to emergency response procedures that use chemicals to protect refugees and displaced persons from vector-borne diseases would also reduce the extent of human and environmental contamination.

Lastly, the ethical issues of treating something bad with something considered not safe, even if it carries differential health benefits, remains to be fully explored for chemical use in IRS. Such an evaluation would be generic for any chemical.

### Other considerations

DDT is normally not the only bioavailable chemical in use in or near homesteads (Bouwman and Kylin 2009; Bouwman et al. 2006; Sereda et al. 2009), and more chemicals for use in IRS may become approved by WHO (2010). The use of insecticides and other chemicals for domestic purposes may also increase, leading to a more complex exposure situation. The effects of the addition of other toxic activities to the homestead mix therefore needs serious consideration. Arguably, the current DDT-protected human population could be the most nonoccupationally deliberately exposed community in the world. This population consists of millions of people experiencing nonintentional but inevitable exposure. As a consequence, the high DDT burdens experienced by these individuals sends a strong message that research on mixture effects and exposure reduction options are urgent.

## Conclusions

With an increasing number of studies showing effects *in vitro* and *in vivo*, and with strong associations between DDT and human disease conditions shown *in situ* (Eskenazi et al. 2009; for other quotes, see Supplemental Material (doi:10.1289/ehp.1002127)], chronic and high exposures to DDT via IRS must be taken seriously. Based on these considerations and concerns, we therefore suggest invoking precaution on DDT used in IRS for malaria control.

In some situations, DDT will provide the greatest achievable health benefit, but arguing that DDT is safe is ignoring the cumulative indications of many studies. The centrist-DDT position, including its recognition of this paradox, seems the only logical and rational conclusion. We have suggested possible ways to manage or address the paradox. A major priority of the centrist position is to use or develop effective alternatives to DDT. The centrist-DDT position remains, however, open to attack from both sides of the spectrum. The debate is likely to continue, but this must not hinder improvement and innovation for a better, safer, and healthier future.
